# Safety of Administration of Vasopressors Through Peripheral Compared to Central Venous Catheters in a Rural Kenyan Hospital: Protocol for a Prospective Observational Cohort Study

**DOI:** 10.2196/81794

**Published:** 2026-03-05

**Authors:** Grace Hezi Gao, Brian Kimani, Linette Lepore, Zahra Aghababa, Hannah Gitura, Michael Kinuthia, Jack Musau, Judy Muthoki, Alice Mwonge, Fredrick Ndibaru, Ann Viola Ndubi, Juliah Ndungu, Kinzi Nixon, Moses Odhiambo, Winslet Okari, Benjamin Thairu, Lilian Vihenda, Rachel M Wojcik, B Jason Brotherton, Kristina E Rudd

**Affiliations:** 1 Department of Critical Care Medicine, University of Pittsburgh Medical Center, 3520 Fifth Ave., Suite 100, Pittsburgh, PA, 15213, United States, 1 512-810-1084; 2 Department of Medicine, AIC Kijabe Hospital, Kijabe, Kenya; 3 Department of Critical Care Medicine, Center for Research, Investigation, and Systems Modeling of Acute Illness (CRISMA), University of Pittsburgh, Pittsburgh, PA, United States; 4 Department of Pediatrics, AIC Kijabe Hospital, Kijabe, Kenya; 5 Department of Research, AIC Kijabe Hospital, Kijabe, Kenya; 6 Department of Medicine, Division of Pulmonary, Allergy, and Critical Care Medicine, University of Pittsburgh, Pittsburgh, PA, United States; 7 Department of Border and Global Health, University of Arizona, Tucson, AZ, United States

**Keywords:** vasopressors, intensive care, critical care, global health, limited resources, shock

## Abstract

**Background:**

The infusion of vasopressors is a standard treatment for shock, and international guidelines recommend administering these medications through central venous catheters (CVCs) due to concerns about potential extravasation and local tissue injury with peripheral intravenous (PIV) administration. However, CVCs are often unavailable in resource-variable settings due to lack of human and material resources. Previous studies have assessed the safety of vasopressor infusion through PIV catheters but have considered only limited patient populations or a short infusion time or have used retrospective designs that may have failed to capture mild complications.

**Objective:**

The primary objective of this study is to observe and describe the incidence of complications among patients receiving vasopressor infusion via PIV administration. The secondary objective is to assess whether the safety of PIV vasopressor administration is noninferior to the safety of administration via CVC.

**Methods:**

This prospective observational study is being conducted at African Inland Church (AIC) Kijabe Hospital, a 360-bed tertiary care teaching hospital in rural Kenya. All patients (adult, obstetric, and pediatric) receiving intravenous vasopressor infusions who are admitted to the intensive care unit or high-dependency unit will be included. Patients will be followed up on twice daily from the start of vasopressor infusion to 72 hours after vasopressor discontinuation or death, whichever occurs first. Demographic, physiological, laboratory, therapeutic, and outcome data will be collected.

**Results:**

Consecutive enrollment began in October 2023 and is ongoing. As of July 2025, we have enrolled 190 patients. We anticipate that the time to enroll the number of patients required to reach our power goal will be 24 months.

**Conclusions:**

This study in a resource-variable setting will allow for more accurate and comprehensive data collection on vasopressor administration and potential complications as they arise, whereas most previous studies have been retrospective in nature. In addition, this is the first study of its kind to include both adult and pediatric patients within a mixed intensive care unit population with broad etiologies of shock, which could improve generalizability.

## Introduction

Shock is a life-threatening condition characterized by inadequate global oxygen delivery relative to demand [1]. It is a common problem in hospitalized patients, can result from various etiologies, most frequently septic shock, and is a leading cause of mortality worldwide [2]. The infusion of vasopressors is a critical component of shock management, with recommendations not to delay use of vasopressor administration until fluid resuscitation is completed but to initiate vasopressors early to achieve target mean arterial pressure [3]. While international guidelines recommend rapid initial administration of these medications through peripheral intravenous (PIV) catheters for a short period if central venous catheters (CVCs) are unavailable, they recommend eventual transition to CVCs for continued administration due to concerns about extravasation and local tissue injury with PIV administration [4].

However, CVCs are often unavailable or challenging to use in many resource-variable settings due to supply chain issues, limited clinician training, and infection control concerns [5]. Recognizing these challenges, several studies have been conducted to assess the safety of vasopressor infusion through PIV catheters as an alternative to CVCs by evaluating the frequency of adverse events of peripherally administered vasopressors [6,7]. A 2019 retrospective single-center cohort study by Lewis et al [8] evaluated 202 patients who received peripheral vasopressors, primarily norepinephrine, with a median infusion duration of 7.5 hours. The incidence of extravasation events was 4%, all managed conservatively. An Australian cohort study of 202 patients reported a 23.2% complication rate with peripheral vasopressor administration, mostly involving phenylephrine infusions for a median duration of 10 hours [9]. In a cancer hospital in India with resources similar to those of the proposed study setting, 122 adults received peripheral vasopressors for a median duration of 11 hours, with only 1 case of extravasation-related tissue injury [10]. A 2022 prospective study in a Pakistani pediatric intensive care unit (ICU) reported a 2.2% extravasation injury rate among 369 patients receiving peripheral vasopressors, primarily epinephrine, for a median duration of 24 hours [11]. A recent prospective cohort study conducted across multiple hospitals in Addis Ababa, Ethiopia, enrolling adults with circulatory shock also found low rates of extravasation injury (3 in 250 patients who received peripheral vasopressors). None of the extravasation injuries occurred in patients who received peripheral vasopressors for less than 5 days [12].

When available, CVCs are not without complications [13]. A study from a high-volume facility in South Africa found a complication rate of 18% among more than 1000 patients receiving a CVC [14]. A recent meta-analysis of 130 studies found a 3% rate of major complications associated with CVC placement—including arterial cannulation or puncture, pneumothorax, infection, and deep vein thrombosis [15]. In the pediatric population, as many as 1 in 4 CVCs fail before completion of therapy [16]. Placement of a CVC is the main risk factor for development of deep vein thrombosis in children, with one study finding an incidence of CVC-related thrombosis of 30.3% [17,18].

Even in well-resourced settings in which CVCs are readily available, there may still be a role for peripheral vasopressor administration. Studies have shown that early initiation of vasopressors in patients with septic shock is associated with increased survival [19]. It generally takes more time to place CVCs as compared to PIV catheters, and depending on the location of the CVC, radiographic confirmation of placement must be obtained before it can be used, further delaying initiation of vasopressors. Thus, multiple previous studies have been conducted to describe and assess the prevalence of complications of peripheral vasopressor administration. A 2020 systematic review and meta-analysis by Tran et al [20], which included 8 observational studies and 1 randomized controlled study with a total of 1835 adult patients who received peripheral vasopressor administration, reported 7% adverse event occurrence, with only 4% of those being major events. Similarly, another systematic review and meta-analysis by Owen et al [21] in 2021, which included both adults and children, reported a 1.8% complication rate for PIV catheters. A 2015 systematic review by Loubani and Green [22], which included primarily case reports, described published reports on local tissue injury or extravasation events. The average duration of peripheral vasopressor administration was 55.9 hours before local tissue injury and 35.2 hours before extravasation [22]. Most recently, a secondary analysis of the Crystalloid Liberal or Vasopressors Early Resuscitation in Sepsis trial evaluated the route of vasopressor initiation and continuation of peripheral vasopressors beyond 6 hours and found that most patients had vasopressors initiated peripherally and continued past 6 hours, with rare low-grade complications [23,24]. Importantly, none of these reports were from the African continent.

Collectively, these studies suggest that short-term peripheral vasopressor administration may be a reasonable alternative when CVCs are unavailable. However, most studies have evaluated relatively limited patient populations and short infusion times. Additionally, most studies have been retrospective, limiting the ability to capture administration and complication details comprehensively. To date, few studies have thoroughly evaluated the safety profile of prolonged peripheral vasopressor infusions regarding duration or concentration, particularly in resource-variable settings in which PIV catheter use is common. Finally, to our knowledge, no study has directly compared the safety of vasopressor administration via PIV catheter vs CVC.

At AIC Kijabe Hospital (KH) in Kenya, it is standard practice to use PIV catheters for the duration of a patient’s vasopressor requirement, often lasting several days, regardless of the agent used or concentration and dose required. There is no maximum concentration or dose for which it is required or standard to switch to a CVC. This is because CVCs are infrequently used due to cost, supply chain issues, and limited clinician training. Therefore, this study aims to address knowledge gaps by documenting the complication rates among patients receiving vasopressor infusion through PIV catheters.

## Methods

### Objectives

The primary objective of this study is to observe and describe the incidence of complications among patients receiving vasopressor infusion via PIV catheter. The secondary objective is to assess whether the safety of vasopressor administration via PIV catheter is noninferior to administration via CVC. This study aims to fill the gap in data on the safety of prolonged peripheral vasopressor administration in resource-variable settings.

### Setting

This is a prospective observational single-center cohort study to be conducted at KH. KH is a rural 350-bed tertiary care teaching hospital that receives referrals from all over Kenya and East Africa. There are a number of graduate medical education programs at KH, including residency programs for physicians, postgraduate clinical officer training in emergency and critical care, and nursing education. Services available at KH include adult medicine, pediatric medicine, obstetrics and gynecology, surgical services, and outpatient clinics. KH has a 5-bed adult ICU, 10 adult high-dependence unit (HDU) beds, a 5-bed maternity HDU (obstetric patients who are critically ill are admitted to the adult ICU), and an 8-bed combined pediatric ICU+HDU. At KH, the HDUs are capable of caring for patients who are critically ill and do not require invasive mechanical ventilation, including those receiving vasopressor infusions. All hospitalized patients receiving vasopressor infusions at KH are admitted to an HDU or ICU and are not admitted to general hospital wards.

### Eligibility Criteria

All adult, maternity, and pediatric patients receiving intravenous (IV) vasopressor (eg, norepinephrine, epinephrine, vasopressin, or dopamine) infusion for less than 24 hours admitted to the ICU or HDU and receiving their infusion via a PIV catheter or CVC placed at KH will be included regardless of the etiology of shock. Post–cardiac arrest patients achieving return of spontaneous circulation and requiring vasopressor infusion to maintain acceptable blood pressure will be included. If a patient experiences cardiac arrest while at KH and was not on vasopressors previously, they will be included if return of spontaneous circulation is achieved and ongoing vasopressor support is required.

Patients not receiving vasopressor infusions will be excluded. Additionally, patients requiring vasopressor support solely while in the operating theater (operating room) or the casualty (accident and emergency department) or dialysis unit will be excluded due to the inability of the study team to maintain adequate data tracking. Individuals transferred to KH with a CVC in situ will also be excluded due to the inability to fully track complications related to CVC placement. Patients in the general maternity, adult, or pediatric acute care wards or the neonatal ICU or those who have received vasopressor infusion for >24 hours before enrollment will also be excluded. Neonates receiving vasopressors via umbilical vein catheter will be excluded. Patients receiving vasopressors via intraosseous access will not be included. However, if they start to receive vasopressors via a PIV catheter or CVC, they will then be eligible for this study. Textbox 1 provides full details of the inclusion and exclusion criteria.

Textbox 1. **Inclusion and exclusion criteria.**

**Inclusion criteria**

- Any adult, pediatric, or maternity patient admitted to an intensive care unit (ICU) or high-dependence unit (HDU) receiving vasopressor (epinephrine, norepinephrine, vasopressin, dopamine, or phenylephrine) infusion
- Vasopressors being administered via peripheral intravenous catheter placed at any facility or via central venous catheter (CVC) placed at African Inland Church Kijabe Hospital
- Vasopressor infusion for <24 hours before screening for study enrollment

**Exclusion criteria**

- Patients not receiving vasopressor infusion
- Patients requiring vasopressor support only while in the operating theater or casualty (eg, accident and emergency department) or dialysis unit
- Patients with cardiac arrest without return of spontaneous circulation
- Patients in maternity, adult, or pediatric wards (outside of ICUs or HDUs)
- Patients in nursery (neonatal ICU)
- Patients with CVC placed at another facility
- Patients receiving only an inotrope (eg, dobutamine) infusion
- Vasopressor infusion for >24 hours before screening for study enrollment

### Patient Enrollment and Data Collection

Eligible patients will be identified and consecutively enrolled by study staff through review of all patients admitted to an HDU or ICU twice every day using the inclusion and exclusion criteria listed in Textbox 1.

A case report form has been created and is available for review (Multimedia Appendix 1). At the time of enrollment, demographic identifiers such as age and gender will be recorded. The types of shock reflected in the electronic medical record on admission will be recorded as the etiology for vasopressor administration. Patient location at the time of enrollment will be recorded, as well as the primary medical service caring for the patient (eg, medicine or surgery). Clinical information such as vital signs and laboratory values will be recorded as the highest and lowest values within 12 hours of enrollment for each variable. Oxygen support (eg, liters per minute) will be recorded, including whether a patient is receiving invasive mechanical ventilation. Finally, patient status at the time of discontinuation of vasopressor (ie, alive vs dead) will be recorded.

Enrolled patients will be followed up on from the start of vasopressor infusion to 72 hours after discontinuation or death, whichever occurs first. Patients will be evaluated twice daily for the occurrence of complications, and any new complications will be documented by study team members.

Information on complications will be recorded in the case report form along with how the complication was addressed (ie, phentolamine vesicant injection, surgical intervention, change of cannula, or no intervention needed). All vasopressors are infused through a syringe pump.

Detailed data on the type, rate, and route of vasopressor infusion will be recorded twice daily on a bedside data collection form (Multimedia Appendix 2). Data obtained from the bedside will include site and route of IV vasopressor infusion, size of PIV cannula, number and name of vasopressors infused, and highest rate of vasopressor infusion during each 12-hour shift. Each vasopressor at KH has a standard concentration regardless of peripheral or central route of administration, which will be used to determine the maximum dose used during vasopressor infusion. The standard concentrations can be found in Table 1. A study team clinician that is participating in the care of each patient will record the maximum rate and IV access information (eg, infusion complications, loss of IV catheter, hypotension, and transition to CVC) for the previous 12 hours. All peripheral cannulas at KH are of standard length—there are no long peripheral cannulas. In addition, all CVCs are standard—no midline catheters or peripherally inserted CVCs are used at KH. All patient care will be delivered according to standard clinical practices at KH and will in no way be altered for study patients.

**Table 1.** Standard concentrations of vasopressors at African Inland Church Kijabe Hospital.

Vasopressor	Concentration
Norepinephrine	8 mg per 50 mL
Epinephrine	8 mg per 50 mL
Vasopressin	20 units per 50 mL
Dopamine	500 mg per 50 mL

Other variables of interest will be hospital mortality, duration of vasopressor infusion, length of hospital stay, and overall disposition. Severity of illness using the Modified Sequential Organ Failure Assessment, Modified Early Warning Score, and Pediatric Logistic Organ Dysfunction score will be calculated. We will collect data regarding CVC placement conditions, including environmental conditions of placement (eg, sterile vs nonsterile and ward vs operating theater), use of ultrasound guidance, and medical service placing the CVC. Duration of CVC placement will also be tracked.

In the event of missing data, we will review the electronic medical record to attempt to obtain these data. All collected data will be entered into a secure electronic database using REDCap (Research Electronic Data Capture; Vanderbilt University). Quality checks will be conducted regularly to ensure the accuracy and completeness of the data. Access to the database will be restricted to authorized personnel only.

### Outcomes

Our primary end point of interest is a composite of any complication of peripheral vasopressor infusion (Textbox 2). Our secondary end point of interest is the comparison of rates of complications among patients receiving vasopressor infusion via PIV catheters vs CVCs (Textbox 3). All complications will be recorded and grouped into mild, moderate, or severe categories for analysis.

Textbox 2. **Adverse events of peripheral vasopressor administration.**

**Mild**

- Extravasation or infiltration
- Thrombophlebitis
- Loss of line
- Blistering

**Moderate**

- Cellulitis
- Skin necrosis or ulcer
- Hypotension due to loss of line

**Severe**

- Gangrene
- Digital ischemia or necrosis

Textbox 3. **Adverse events of central vasopressor administration.**

**Mild**

- Difficult line placement documented by the clinical team
- Arterial puncture
- Unplanned line removal (eg, fell out or patient pulled it out)
- Thrombosis of line
- Thrombophlebitis

**Moderate**

- Line-associated deep venous thrombosis
- Hypotension due to loss of line

**Severe**

- Pneumothorax
- Associated bloodstream infection

### Sample Size and Power Calculations

Assuming approximately 4:1 enrollment of patients receiving vasopressor infusions via PIV catheters vs CVCs (consistent with baseline rates at KH), the necessary sample size to meet all study objectives is 143. Our primary objective is descriptive in nature, so power calculations are not applicable. For our secondary objective (test of noninferiority), we assumed that the complication rates of CVC and PIV vasopressor administration are 25% and 10%, respectively, based on clinical experience at KH and literature review. With an absolute tolerable difference of up to 10%, we calculated that we need a sample size of 29 patients in the CVC group and 114 in the PIV group to assess noninferiority with 80% power and a one-sided α of .025.

### Data Analysis Plan

Statistical analyses will be performed on all patients enrolled using the Stata analytical software (version 16; StataCorp). Categorical data will be described using percentages, whereas continuous data will be described using median values and IQRs as we anticipate potential nonnormal distributions due to the nature of the variables. Chi-square tests will be conducted for categorical data, and Wilcoxon rank sum tests will be conducted for continuous data. Statistical significance will be determined at *P*<.05. There are planned, prespecified subgroup analyses for adults (aged ≥16 years) and children (aged ≤15 years). Models will be adjusted for baseline characteristics where appropriate.

### Ethical Considerations

This study has received ethics approval from the KH and University of Pittsburgh institutional review boards (approval numbers: KH/ISERC/02718/0060/2023; STUDY23080034). It will be conducted under a waiver of consent as it is a noninterventional study with minimal risk that does not deviate from the current standard of care. There are no planned interventions, nor are there plans to change standard practices at KH. This study does not plan to prohibit the insertion of CVCs in patients with shock. The decision to do so will be made at the judgment of the treating clinician. Privacy and confidentiality of research participant data and identity will be maintained throughout enrollment and data collection.

### Dissemination

In accordance with international research standards, we will share the results of this study as widely as possible, with particular attention to disseminating the findings with the clinical staff at KH in each unit where we conduct the study. We will also share our results with the international medical community by submitting at least one full-length manuscript for publication in a peer-reviewed open access journal and submitting abstracts to relevant scientific conferences. When possible, we will give priority to conferences in Kenya or sub-Saharan Africa, with special attention to AIC Kijabe Research Day.

## Results

Consecutive enrollment began in October 2023 and is currently ongoing. We anticipate that the time to enroll the number of patients required to reach our power goal will be 24 months. Once this goal has been reached, we estimate that reviewing, analyzing, and filling in the missing data will take 6 additional months. During this time, there will be periodic review of the data collected to ensure accuracy, as well as periodic analysis to assess for the occurrence of our primary outcome.

## Discussion

This is a prospective, investigator-initiated, single-center observational cohort study that plans to enroll and follow up on patients receiving vasopressor infusions via PIV catheters and CVCs to describe and compare the incidence of complications. Our hypothesis is that there will be few severe complications of administration of vasopressors via PIV catheter in this setting and that the safety of vasopressor administration via PIV catheter will be noninferior to that of administration via CVC.

This study is being conducted at a rural Kenyan hospital in a resource-variable setting. KH was chosen as the study site for a variety of reasons. Most importantly, the current usual practice at KH is the use of PIV catheters for administration of vasopressors regardless of agent, duration, or rate used. CVCs are not commonly placed primarily for vasopressor administration but, rather, are more commonly placed for other indications, such as total parenteral nutrition, hemodialysis, or chemotherapy, and are sometimes subsequently used for vasopressors if available [25]. This is in contrast with current standard practice in many high-resource settings, where vasopressor administration is frequently a primary indication for CVC placement. In addition, the duration of vasopressor administration via PIV catheter at KH can be multiple days. This differs from current international guidelines that recommend insertion of a CVC to continue vasopressor administration for prolonged infusion times (>6 hours) [4]. Thus, KH provides a unique setting for this study to help provide evidence on the adequacy and safety of peripheral vasopressor administration in resource-variable settings in which CVC placement may not be feasible.

Compared to previous studies, this is the first study to include both adult and pediatric patients, resulting in a relatively large sample size. In addition, patients are enrolled not just from the ICU but also from the HDU, and they include a mixed ICU population with broad etiologies of shock not limited to septic shock, which improves the generalizability of the results. As mentioned previously, many patients at KH have a prolonged duration of vasopressor infusion via PIV catheter. This contrasts with previous studies that have mostly looked at short infusion times, which not only provides an ideal setting for this study but also reflects the reality of resource-variable setting practice. Finally, this is a prospective observational study with enrollment ongoing in real time, allowing for more accurate and comprehensive data collection on vasopressor administration and potential complications as they arise.

There are several limitations to this study, the most important of which is that, as this is a prospective observational study without randomization, there will be residual confounding variables and differences in baseline between the PIV and CVC groups. As patient populations receiving CVCs generally have higher severity of illness and mortality than those without CVCs in critical care studies, we will not compare the PIV and CVC groups based on these clinical outcomes. In addition, as there is no blinding, a heightened awareness of potential complications may result in a lower threshold to detect a complication and change the IV site, thereby overestimating the complication rate. We have categorized the complications as mild, moderate, and severe to better account for this. Finally, this study is being conducted in a resource-limited hospital with a limited electronic medical record, with potential for limited or missing data.

This study aims to address the critical knowledge gap in understanding vasopressor administration safety in resource-variable settings in which central venous access may be limited or unavailable. By studying vasopressor delivery in a setting in which PIV catheter use is the primary method of delivery rather than the exception, we hope to provide insights into alternative approaches to critical care medication delivery in resource-variable settings.

## Supplementary material

10.2196/81794 Multimedia Appendix 1 Case report form.

10.2196/81794 Multimedia Appendix 2 Bedside data collection sheet.

## Abbreviations

AIC: African Inland Church
CVC: central venous catheter
HDU: high-dependence unit
ICU: intensive care unit
IV: intravenous
KH: African Inland Church Kijabe Hospital
PIV: peripheral intravenous
REDCap: Research Electronic Data Capture
