# Supplementary material for: Safety of Administration of Vasopressors Through Peripheral Compared to Central Venous Catheters in a Rural Kenyan Hospital: Protocol for a Prospective Observational Cohort Study
Source: JMIR Res Protoc. 2026 Mar 5;15:e81794. doi: 10.2196/81794 (PMC12978963; doi:10.2196/81794)
Supplement: Multimedia Appendix 1 [file resprot-v15-e81794-s001.docx]

**S1 Appendix 1 - Case Report Form**

**Hospital IP Number**

**OTHER IDENTIFYING INFORMATION - To be completed at time of study enrollment**

Age ☐ Days ☐ Weeks ☐ Months (if <24 months old) ☐ Years

Gender ☐ Male ☐Female

Is the patient on a vasopressor at the time of study enrollment? ☐ Yes ☐No

If yes, route of infusion at time of enrollment: ☐ Peripheral ☐Central

Type of Shock upon enrollment (obtain from clinician diagnosis in daily note in HMIS, check all that apply)

☐ Septic

☐ Non-septic distributive (e.g., anaphylaxis, neurogenic, adrenal insufficiency, post-arrest, pancreatitis, acute liver failure)

☐ Hemorrhagic

☐ Hypovolemic

☐ Cardiogenic

☐ Undifferentiated or other

Site at time of enrollment

☐ Adult ICU

☐ Adult HDU

☐ Paediatric ICU

☐ Paediatric HDU

☐ Maternity HDU

Primary service

☐ Medical patient

☐ Surgical patient

☐ Obstetric patient

**Hospital IP Number**

**To be completed at time of study enrollment***

Date (*DD/MM/YYYY*) and Time (HH:MM AM/PM) of Arrival to Kijabe hospital

Date (*DD/MM/YYYY*) and Time (HH:MM AM/PM) of Enrollment

Temp Highest in last 12hr Lowest in last 12hr

HR Highest in last 12hr Lowest in last 12hr

RR Highest in last 12hr Lowest in last 12hr

SBP Highest in last 12hr Lowest in last 12hr

MAP Highest in last 12hr Lowest in last 12hr

GCS Highest in last 12hr Lowest in last 12hr

AVPU** Highest in last 12hr Lowest in last 12hr

SpO2 Highest in last 12hr Lowest in last 12hr

FiO2 ☐ Room air ☐ L/min

Intubated? ☐ Y ☐ N

PaO2 (if available) Highest in last 12hr Lowest in last 12hr

PaCO2 or PvO2 (if available) Highest in last 12hr Lowest in last 12hr

Scleral icterus or jaundice at time of enrollment? ☐ Y ☐ N

Pupil response? ☐ Both responsive ☐ Both fixed ☐ Other

Serum creatinine Highest in last 12hr Lowest in last 12hr

WBC count Highest in last 12hr Lowest in last 12hr

Platelet count Highest in last 12hr Lowest in last 12hr

Lactate Highest in last 12hr Lowest in last 12hr

*This information is to be obtained from HMIS. Where indicated, the highest and lowest values within 12 hours of enrollment are to be written in the space provided.

**If AVPU is used instead of GCS, then record the highest (e.g., “A”) or lowest (e.g., “U”) value during the 12 hours preceding enrollment. This will be converted to a corresponding GCS number.

**Hospital IP Number**

**OUTCOMES - To be completed within 24hr of vasopressor discontinuation**

Was the patient alive at discontinuation of vasopressor? ☐ Yes ☐ No

Was a CVC ever placed at any time from admission up to discontinuation of vasopressors? If yes, please ensure page 5 is completed for each new CVC.

☐ Yes ☐ No

**OUTCOMES - To be completed at 72hr after vasopressor discontinuation**

Was there any complication of vasopressors (see page 4 for complete list) up to 72hr after discontinuation of vasopressors? If yes, please ensure page 4 is completed for each complication.

☐ Yes ☐ No

**OUTCOMES - To be completed upon discharge**

Discharge Date (DD/MM/YYYY)

Disposition

☐ Home

☐ Transfer to Other Facility

☐ Death, Cause of Death (per HMIS) ____________

Discharge diagnoses (per HMIS)

1. ______________________________
2. ______________________________

Did the patient have a complication of vasopressors documented up to 72hr after discontinuation of vasopressors? If yes, at any time during hospitalization, did the patient have one of the following treatments for their complication: ☐ None ☐ Surgical debridement ☐ Amputation ☐ Other____________________

Did the patient ever have vasopressors administered through a CVC? If yes, did they ever have a positive blood culture during the hospitalization: If yes,

Date(s) that positive blood culture was drawn (DD/MM/YYYY)____________________

Date(s) that positive blood culture was drawn (DD/MM/YYYY)____________________

Date(s) that positive blood culture was drawn (DD/MM/YYYY)____________________

**Hospital IP Number**

**Complication Information: To be reviewed each day through 72hr after vasopressor discontinuation by study team member and completed as soon as possible after each new complication. If no complications occur within 72hr of stopping vasopressors, leave this page blank.**

Date of complication (DD/MM/YYYY): _____________

Complication of peripheral vasopressor use:

☐ Extravasation/Infiltration ☐ Digital ischemia/necrosis

☐ Loss of line ☐ Hypotension due to loss of line

☐ Thrombophlebitis ☐ Blistering

☐ Gangrene ☐ Skin necrosis/ulcer

☐ Cellulitis

Complications of central venous catheter vasopressor use:

☐ Associated bloodstream infection

☐ Unplanned line removal (e.g., fell out or patient pulled out)

☐ Hypotension due to loss of line

☐ Thrombosis of line

☐ Line-associated deep venous thrombosis

☐ Thrombophlebitis

☐ Difficulty with line placement

☐ Arterial puncture

☐ Pneumothorax

How was complication addressed:

☐ Vesicant given ☐ Surgical review ☐ Surgical intervention

☐ No intervention required ☐ Other

Was a new line inserted for continuation of vasopressor ☐ Yes ☐ No

If yes, what type of line ☐ Peripheral ☐Central

Was there a new complication with the new line ☐ Yes ☐ No

If yes, fill out a new complication form (page 4).

**Hospital IP Number**

**Central venous catheter (CVC) information - To be reviewed each day through time of vasopressor discontinuation by study team member and completed as soon as possible after each new CVC is placed. If no CVC is placed during that time, leave this page blank.**

Date central venous catheter placed (DD/MM/YYYY): _____________

Service that placed central venous catheter: ☐ Surgical clinician ☐ Medical clinician

How was the CVC placed? ☐ Emergent bedside - nonsterile ☐ Bedside - sterile ☐ Theatre - sterile

Was ultrasound guidance used?: ☐ Yes ☐ No

Indication for central venous catheter insertion:

☐ Loss of peripheral catheter ☐ Escalating pressor doses

☐ To administer a medication other than vasopressor (e.g., TPN, chemotherapy)

☐ Clinician preference ☐ Not documented

Complications of central venous catheter insertion:

☐ Pneumothorax ☐ Arterial puncture

☐ >3 needle sticks ☐ Arrhythmia

Site of central venous catheter insertion:

☐ Internal jugular vein ☐ Subclavian vein ☐ Femoral vein ☐ Umbilical vein

**To be reviewed daily and completed once CVC is removed:**

Date central venous catheter removed (DD/MM/YYYY): _________________
