# Supplementary material for: Safety of Administration of Vasopressors Through Peripheral Compared to Central Venous Catheters in a Rural Kenyan Hospital: Protocol for a Prospective Observational Cohort Study
Source: JMIR Res Protoc. 2026 Mar 5;15:e81794. doi: 10.2196/81794 (PMC12978963; doi:10.2196/81794)
Supplement: Multimedia Appendix 2 [file resprot-v15-e81794-s002.docx]

**S2 Appendix 2 - Bedside Data Collection Sheet**

**Hospital IP Number**

***Please document this information at the end of your respective shift.***

| Today’s Date  (DD/MM/YY) |  | |  | |  | |  | |
| --- | --- | --- | --- | --- | --- | --- | --- | --- |
|  | DAY | NIGHT | DAY | NIGHT | DAY | NIGHT | DAY | NIGHT |
| During your shift was the patient ever on a vasopressor (Y/N)? |  |  |  |  |  |  |  |  |
| Does your patient have a central line (Y/N)? |  |  |  |  |  |  |  |  |
| **Vasopressor 1 name** (see key) |  |  |  |  |  |  |  |  |
| Vasopressor 1 Max rate over shift (**mL/hr**) |  |  |  |  |  |  |  |  |
| Was vasopressor 1 newly started during your shift? If yes, time started (HH:MM AM/PM) |  |  |  |  |  |  |  |  |
| Was vasopressor 1 stopped during your shift? If yes, Time stopped (HH:MM AM/PM) |  |  |  |  |  |  |  |  |
| Vasopressor 1 Cannula type (PIV vs CVC; *document only the most recent cannula if >1 during shift)* |  |  |  |  |  |  |  |  |
| If PIV, vasopressor 1 Cannula size (*document only the most recent cannula if >1 during shift)* |  |  |  |  |  |  |  |  |
| Vasopressor 1 Cannula site (*document only the most recent cannula if >1 during shift)* |  |  |  |  |  |  |  |  |
| Were there any changes to cannula type, size, or site during your shift (Y/N)? |  |  |  |  |  |  |  |  |
| **Vasopressor 2 name** (see key) |  |  |  |  |  |  |  |  |
| Vasopressor 2 Max rate over shift (**mL/hr**) |  |  |  |  |  |  |  |  |
| Was vasopressor 2 newly started during your shift? If yes, time started (HH:MM AM/PM) |  |  |  |  |  |  |  |  |
| Was vasopressor 2 stopped during your shift? If yes, Time stopped (HH:MM AM/PM) |  |  |  |  |  |  |  |  |
| Vasopressor 1 Cannula type (PIV vs CVC; *document only the most recent cannula if >1 during shift)* |  |  |  |  |  |  |  |  |
| If PIV, vasopressor 2 Cannula size (PIV vs CVC; *document only the most recent cannula if >1 during shift)* |  |  |  |  |  |  |  |  |
| Were there any changes to cannula type, size, or site during your shift (Y/N)? |  |  |  |  |  |  |  |  |
| Today’s Date  (DD/MM/YY) |  | |  | |  | |  | |
|  | DAY | NIGHT | DAY | NIGHT | DAY | NIGHT | DAY | NIGHT |
| **Vasopressor 3 name** (see key) |  |  |  |  |  |  |  |  |
| Vasopressor 3 Max rate over shift **(mL/hr)** |  |  |  |  |  |  |  |  |
| Was vasopressor 3 newly started during your shift? If yes, time started (HH:MM AM/PM) |  |  |  |  |  |  |  |  |
| Was vasopressor 3 stopped during your shift? If yes, Time stopped (HH:MM AM/PM) |  |  |  |  |  |  |  |  |
| Vasopressor 3 Cannula type (PIV vs CVC; *document only the most recent cannula if >1 during shift)* |  |  |  |  |  |  |  |  |
| If PIV, vasopressor 3 Cannula size (*document only the most recent cannula if >1 during shift)* |  |  |  |  |  |  |  |  |
| Vasopressor 3 Cannula site (*document only the most recent cannula if >1 during shift)* |  |  |  |  |  |  |  |  |
| Were there any changes to cannula type, size, or site during your shift (Y/N)? |  |  |  |  |  |  |  |  |

**Chart key to be used for documentation:**

*Vasopressor: NE – norepinephrine; EP – epinephrine, VA – vasopressin; DO – dopamine*

*Site: H – hand; F – forearm; AC – antecubital fossa; U – upper arm; EJ – external jugular; IJ – internal jugular; FM – femoral vein; S - Subclavian; LL - lower extremity; UV - umbilical vein*

*Size: 16g, 18g, 20g, 22g, 24g, 25g*

*CVC (central venous catheter)*
